# Semantic Segmentation of Natural Materials on a Point Cloud Using Spatial and Multispectral Features

**DOI:** 10.3390/s20082244

**Published:** 2020-04-15

**Authors:** J. M. Jurado, J. L. Cárdenas, C. J. Ogayar, L. Ortega, F. R. Feito

**Affiliations:** Computer Graphics and Geomatics Group of Jaén, University of Jaén, 23071 Jaén, Spain

**Keywords:** multispectral imaging, heterogeneous data fusion, point cloud segmentation, material-based recognition

## Abstract

The characterization of natural spaces by the precise observation of their material properties is highly demanded in remote sensing and computer vision. The production of novel sensors enables the collection of heterogeneous data to get a comprehensive knowledge of the living and non-living entities in the ecosystem. The high resolution of consumer-grade RGB cameras is frequently used for the geometric reconstruction of many types of environments. Nevertheless, the understanding of natural spaces is still challenging. The automatic segmentation of homogeneous materials in nature is a complex task because there are many overlapping structures and an indirect illumination, so the object recognition is difficult. In this paper, we propose a method based on fusing spatial and multispectral characteristics for the unsupervised classification of natural materials in a point cloud. A high-resolution camera and a multispectral sensor are mounted on a custom camera rig in order to simultaneously capture RGB and multispectral images. Our method is tested in a controlled scenario, where different natural objects coexist. Initially, the input RGB images are processed to generate a point cloud by applying the structure-from-motion (SfM) algorithm. Then, the multispectral images are mapped on the three-dimensional model to characterize the geometry with the reflectance captured from four narrow bands (green, red, red-edge and near-infrared). The reflectance, the visible colour and the spatial component are combined to extract key differences among all existing materials. For this purpose, a hierarchical cluster analysis is applied to pool the point cloud and identify the feature pattern for every material. As a result, the tree trunk, the leaves, different species of low plants, the ground and rocks can be clearly recognized in the scene. These results demonstrate the feasibility to perform a semantic segmentation by considering multispectral and spatial features with an unknown number of clusters to be detected on the point cloud. Moreover, our solution is compared to other method based on supervised learning in order to test the improvement of the proposed approach.

## 1. Introduction

The precise observation of three-dimensional (3D) natural spaces is a research line in which many recent works have proposed novel solutions with a high impact in the science of materials and remote sensing [1]. The emergence of a wide variety of sensors makes it possible to develop detailed studies about the recognition of meaningful features of every entity in nature. In an ecosystem there are many living organisms and nonliving objects, which are influenced by several environmental effects. The characterization of this scenario by the 3D reconstruction of plants, rocks or trees, as well as the capture of light interactions between them play a key role to gather comprehensive data of the environmental sustainability. However, nature tends to be chaotic and the recognition of all existing entities in a natural space is not a trivial task. These objects usually present a similar appearance in the visible range and can be occluded by some overlapping structures. In this work, we focus on the extraction and fusion of spectral and spatial properties of a real-life scenario for the semantic segmentation of some natural entities, which are characterized by different materials. Many remote sensing application and monitoring tasks will benefit from having access to the proposed method for the material segmentation in a natural environment. For instance, the creation of a forest inventory, the object recognition to study the ground composition, the survival of a plant species or the impact of environmental pollution may use the proposed material-based segmentation in this research.

The modeling of 3D structures in natural scenarios has been improved through the proposal of recent approaches based on different techniques such as RaDAR [2], Light Detection and Ranging (LiDAR) [3] or structure-from-motion (SfM) [4]. Regarding the improvement of the camera resolution, the use of image-based methods for 3D reconstruction has increased because these are based on a more cost-effective solution than costly LiDAR systems. The potential of close-range photogrammetry (CRP) to produce dense and accurate point clouds has increased in recent years [5,6]. The resulting 3D models have sufficient geometric details to depict the 3D shape of objects captured. SfM-based [7] methods enable the creation of geometrically precise point cloud datasets based entirely on large sets of overlapping images taken from multiple viewpoints. Recent research has been presented for the 3D reconstruction of urban and natural environments by using conventional cameras [8,9]. Instead of managing a triangular mesh, which requires an additional computational cost, the point clouds are widely used to provide an accurate representation of existing objects in nature. This geometric characterization is a great opportunity to extract morphological features and to discover the spatial distribution of every entity in this complex scenario. The use of 3D models instead of the image segmentation of materials is one of the contributions of this work. However, the semantic recognition among homogeneous materials in vegetation areas, where many structures also present a similar shape, is challenging. In addition to the use of spatial features and the visual appearance to classify different natural materials, the multispectral sensors provide extra channels to measure the lighting interactions between every entity of the environment.

Multispectral imaging provides meaningful features to differentiate similar materials whose reflectance significantly changes in a specific narrow band [10]. Lighting is one of the most important factors to study the visual appearance of materials. The observation of the object reflectance by multispectral images is a key technique to model the physical process of light interacting with materials [11]. Moreover, the multispectral images have been used for the shadow removal in urban spaces [12] and image segmentation of materials [13,14]. In the field of computer vision, some previous works propose different approaches for plant phenotyping considering the spectral response [15], the recognition of different geological formations using a multispectral camera [16] and the detection of urban materials [17,18]. Other recent approaches have proposed a semantic classification of urban spaces by applying neural networks [19,20] or using LiDAR data and a clustering approach [21]. Furthermore, the use of deep learning techniques for unsupervised scenarios is a promising research [22,23]. The automatic recognition of homogeneous materials in a natural space, which contains a high diversity of objects, is one of the fundamental problems of computer vision. This issue is posed in this paper by observing the spectral reflectance on the 3D structure of the studied scenario.

The acquisition of multispectral images is useful to get a comprehensive knowledge of light interactions in natural environments. Recent methods propose solutions using hyperspectral, multispectral and thermal images for the environmental understanding. The merge of geometric properties with other data types like the spectral reflectance reveal some feature patterns, which cannot be easily detected in the visible range. Several studies have proposed different solutions by considering heterogeneous image datasets to extract a comprehensive knowledge of the development of ecosystems. Nevalainen et al. [24] used hyperspectral imaging for an individual tree detection with UAV-based photogrammetric point clouds and Degerickx et al. [25] provided an urban tree health assessment using airborne hyperspectral and LiDAR imagery.

The proposed method aims to extract different types of materials from a 3D model of nature. The study scenario is characterized by the homogeneity of materials, so it is difficult to differentiate them. Our approach is based on the characterization of the 3D model geometry with multispectral data for the semantic segmentation of materials. Therefore, high resolution RGB images and multispectral images have been taken at the same time. Both sensors have been integrated into the same system designed by our team.

This article is structured as follows: Section 2 describes the study scenario and the main methods used for data acquisition, fusing and segmentation. In Section 4, the results are presented and discussed. Finally, Section 6 presents the main conclusions and further research.

## 2. Materials and Methods

**Figure 1:**
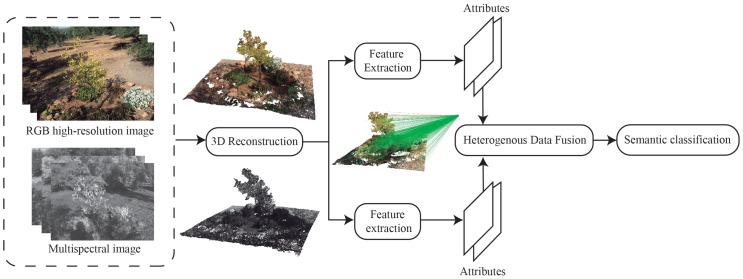
The flow diagram of the proposed methodology.

The study area characterization, the description of the used sensors and the methods applied to acquire, process, and identify the target materials in the studied natural space are presented in this section. The methodology is based on three main stages: (1) multispectral image mapping on the point cloud, (2) feature extraction and (3) semantic segmentation. Figure 1 presents the main steps of the proposed methodology.

### 2.1. The Surveyed Area and Acquisition Process

In this work, we focus on a reduced real-life environment which has been modeled with high detailed geometry. This scenario is characterized by a tree, low vegetation of different species, flowering plants, rocks and rocky ground. Our goal is to identify singular materials in the natural space, which determine a different appearance of the existing entities. Initially, we do not have any previous knowledge about the objects captured in the scene.

**Figure 2:**
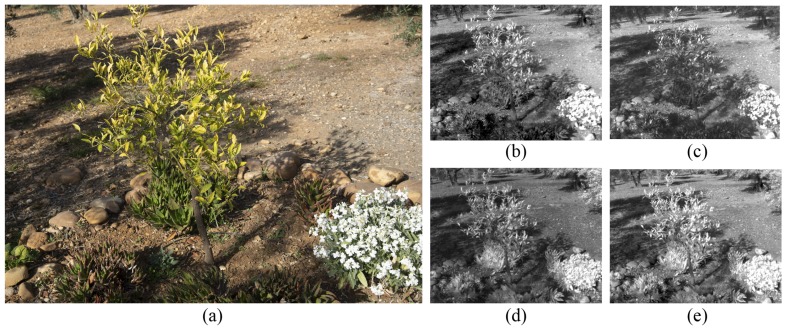
The captured images: (**a**) RGB image, (**b**) green, (**c**) red, (**d**) NIR and (**e**) REG.

According to the acquisition process, a high-resolution RGB camera and a multispectral sensor have been used to capture multiple overlapping images of the studied scenario. Moreover, an Inertial Measurement Unit (IMU) and a Global Positioning System (GPS) is used for georeferencing the RGB images and getting a more accurate 3D reconstruction. Firstly, the full-frame RGB camera (model: Sony Alpha 7 RIII) takes images with 48 megapixels, thereby observing the target scene by a high spatial resolution. These images are georeferenced by using an external IMU/GPS to determine the position and orientation of the camera. Moreover, the external parameters of the camera are set before the acquisition process in order to ensure a correct capture from all viewpoints (ISO: 100, exposure time: 1/200 and f-number: 8). Secondly, the multispectral sensor (model: Parrot Sequoia) is composed by four lenses in order to capture the reflected radiation on different wavelengths and a sunshine sensor to correct the irradiance incident on the study area. Consequently, for each capture four multispectral images are taken. The observed spectral range is the near-infrared (NIR) from 770 nm to 810 nm, red (640–680 nm), green (530–570 nm) and red-edge (REG) from 730 nm to 740 nm. The multispectral images (1.2 megapixels) present a lower resolution than RGB images and higher geometric distortion due to the wide-angle lenses with a focal length of 4mm. The position and orientation of every image is determined by the GPS and IMU, which take part of the multispectral sensor. Figure 2 shows the observed RGB and multispectral bands in the surveyed scenario.

**Figure 3:**
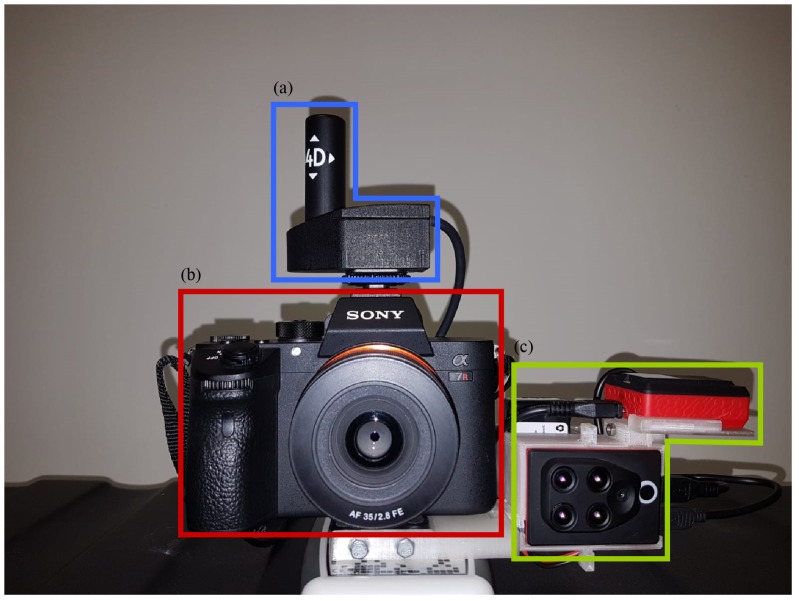
The acquisition system. (**a**) the IMU and GPS, (**b**) the high-resolution camera; and (**c**) the multispectral sensor.

The integration of sensors, described above, is required for a simultaneous capture of high-resolution RGB and multispectral images. For this purpose, a specific object has been produced by 3D printing in order to create a custom camera rig (Figure 3). As a result, the high-resolution camera and the external GPS/IMU, as well as the multispectral sensor can be used together to model the 3D structure of the target scene and to observe the reflectance response of every object of the scene captured.

According to the previous acquisition system, multiple overlapping images have been taken by the RGB camera and the multispectral sensor around the study scenario. On the one hand, 82 RGB images are capture by the high-resolution camera. On the other hand, 82 captures are taken by the multispectral sensor and for each one four spectral bands are observed, so 328 multispectral images are acquired. The overlap between all images is 85% in order to get detailed geometry with a high spatial resolution.

### 2.2. 3D Reconstruction

The 3D modeling of natural environments is not a simple task due to the high complexity of the plant structures and the high occlusion between every object. In nature, many entities are usually overlapped in the same space. Therefore, the correct reconstruction of this type of scenarios requires some considerations. The SfM relies on visual similarities between overlapping images to reconstruct the model. The vegetation has a complex geometry (multiple branches and leaves), which is why it is more difficult to find enough similarities between overlapping images and many images cannot be correctly calibrated. Consequently, it is recommended to set a high overlap between the captured images (upper than 70%) and reduce the image scale on 1/2 for the search of key points in the image.

In this work, the photogrammetric processing is applied through the application of the SfM algorithm [26]. It is an open-source method, which can detect the same regions of overlapping images, determine their geometric relationships and infer the rigid scene structure (point set) with the pose (position and orientation) of all cameras. The resulting 3D structures are modeled as a point cloud instead of a 3D mesh due to the fact that many complex geometric objects cannot be correctly triangulated. Consequently, only the point cloud is the comprehensive 3D model of the study scenario for the extraction of different existing materials. As a result, two point clouds are generated by using RGB and multispectral images. The RGB point cloud has a higher spatial resolution and the Ground Sampling Distance (GSD) is 0.07 cm. The size of the densified point cloud is 8,394,979. Otherwise, the quality of multispectral images is worse than RGB images due to high geometric distortion and lower image resolution. Consequently, the GSD of the resulting point cloud is 0.97 cm. This temporary point cloud is only used for the data alignment and thus, the initial pose of multispectral cameras is corrected by mapping them on the RGB point cloud. This process is explained in Section 2.4.

### 2.3. Multispectral Image Processing

Multispectral imaging provides image information in spectral as well as spatial domains. As mentioned before, various methods have been presented to study the reflectance properties for each natural material by considering different lighting conditions. In natural environments there are similar materials, which characterize different entities. The physiological properties of vegetation play a significant role in the material appearance [27]. The surface bidirectional reflectance distribution function (BRDF) measures the distribution of the reflected light by leaves, which is directly determined by the plant status. Therefore, the mean reflectance of an object is a key feature to find out its corresponding material.

In this work, a multispectral sensor is used to measure the spectral reflectance in a real-life scenario from multiple viewpoints. As mentioned before, this sensor is composed by a camera, which captures the reflected irradiance, and a sunshine sensor, which takes the incident irradiance. The resulting multispectral images provides an accurate measurement of reflectance in some specific narrow-band. Firstly, in the near-infrared (NIR), the vegetation can be easily recognized due to that fact that this band is less sensitive to chlorophyll. Secondly, the green and red bands are very useful to classify similar natural materials according to the light absorption in the visible range. Finally, the REG band captures the reflectance between the red and NIR, so it plays a key role to detect a key contrast from the visible to infrared light.

The capturing spectral reflectance is influenced by several parameters such as changes of ambient light, the shutter speed, etc. Therefore, the multispectral images have to be radiometrically corrected by a calibration target. In our study, the spectral reflectance is calculated by measuring the incoming sunlight irradiance and reflected irradiance by the surface of the object captured. Equation (1) is used to estimate the reflectance value of every pixel for each multispectral image.

(1) $$R=K\left(\frac{\Phi_{e}^{r}}{\Phi_{e}^{i}}\right)\cos\left(\theta \right)$$

where $\Phi_{e}^{r}$ is the radiant flux reflected by the object captured, $\Phi_{e}^{i}$ is the radiant flux incidence by the sun and θ is the angle between the direction vector of sun rays and the direction vector of the sunshine sensor.

The incoming sunlight irradiance ($\Phi_{e}^{i}$) is measured by the sunshine sensor, which is mounted on top of the acquisition system. This device is continuously capturing the lighting conditions during acquisition process. Moreover, the angle between the sunshine sensor and the sunlight direction must be considered to compensate the light reflection. A mean value of the incident light is estimated for each multispectral image. The mathematical formulation of this magnitude is defined in Equation (2).

(2) $$\Phi_{e}^{i}=\frac{\nu }{g\tau }$$

where ν is a sensor count value, *g* is the relative gain factor and τ is the exposure time in seconds.

The next step is to calculate the reflected irradiance ($\Phi_{e}^{r}$) by using metadata (Exif) stored in the image. Every pixel **p** in the image **I** provides a reflected irradiance value, which is calculated by applying the Equation (3).

(3) $$\Phi_{e}^{r}=f^{2}\frac{\rho -B}{A\gamma \varepsilon +C}$$

where *g* is the f-number = 2.2, *p* is defined by pixel intensity, ε is the exposure time, γ is the ISO parameter = 100 and A, B and C are the calibration coefficients measured per camera in production.

Finally, the plant reflectance has to be radiometrically calibrated. Therefore, a calibration panel is used to calculate the calibration coefficient (*K*) regarding a known reflectance for every multispectral band. Three images are captured over this panel with different exposure levels. This process is carried out on the beginning and at the end of the acquisition process. Then, the Equation (4) is applied to calculate a calibration coefficient *K* for each observed band.

(4) $$K_{i}=R_{i}\left(\frac{\Phi_{e}^{i}}{\Phi_{e}^{r}}\right)$$

where $R_{i}$ is the known reflectance for each band *i*, $\Phi_{e}^{i}$ is the radiant flux incidence from the sunlight and $\Phi_{e}^{r}$ is the radiant flux reflected by the calibration panel.

Moreover, the reflectance values in the red and NIR bands are combined to estimate the Normalized Difference Vegetation Index (NDVI) [28]. This spectral index is widely used in remote sensing for the assessment of a plant health. Although this study is beyond the scope of this work, the use of NDVI is very interesting to identify the vegetation on the scene and classify different plant classes. As a result, a reflectance and NDVI maps are obtained for each multispectral image and then mapped on the point cloud to characterize it with meaningful spectral traits.

### 2.4. Heterogeneous Data Fusion

In this section, we describe the process to enrich the RGB point cloud using the spectral reflectance in order to get a more detailed knowledge of the behavior of all existing materials. This approach is based on our previous work presented by Jurado et al. [29]. Consequently, the comprehensive 3D model can also contain multispectral features by mapping the reflectance and NDVI maps on the geometry. For this purpose, the proposed method is divided in two processes: (1) the data alignment and (2) the inverse 3D projection.

The alignment of multispectral images and the high-resolution RGB point cloud is carried out by the development of an automatic method to set the same coordinate system for both datasets. To ensure a fully automated multispectral image mapping, we have applied the iterative-closest-point (ICP) algorithm [30] for the geometry-based alignment of multispectral and RGB point clouds. This method assesses the corresponding point pairs based on computing a weighted average from calculation of distance and compatibility of normal vectors. A normal vector defines how a surface responds to lighting. The amount of light reflected by a surface is determined by its normal vector and the direction of incoming light. These normals are directly calculated at the reconstruction step. As a result, a rigid transformation is obtained minimizing the sum of the squared error in Equation (5).

(5) $$E\left(R,t\right)=\frac{1}{N_{p}}\sum_{i=1}^{N_{p}}\left|\right|x_{i}-Rp_{i}-t{\left|\right|}^{2}$$

where: $x_{i}$ and $p_{i}$ are corresponding points, *t* is the translation vector and *R* is the rotation matrix.

**Figure 4:**
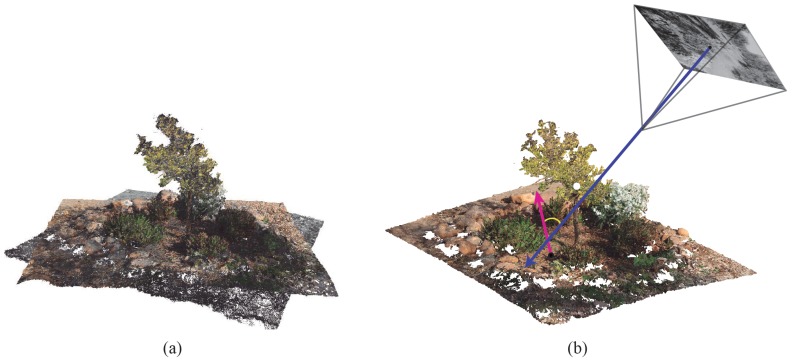
Data Fusion: (**a**) the point cloud alignment; and (**b**) multispectral image mapping on the point cloud.

Then, the inverse projection is developed to determine the matching between the 3D point and its corresponding pixel in the multispectral image. Figure 4 shows the point cloud and main vectors, which are considered for the image mapping. The blue arrow is the direction vector of the camera and the purple arrow is the normal vector of a 3D point. The reflectance values of a multispectral image are weighted during the mapping process by considering the angle between both vectors.

By applying our method every 3D point (with coordinates: *X, Y, Z*) is mapped to image coordinates ($x_{d}$, $y_{d}$) by considering the fisheye model of the multispectral camera. This distortion model is determined by the parameters C, D, E and F, which describe an affine deformation of the circular image in pixel coordinates. The polynomial fisheye, with the coefficients $p_{2}$, $p_{3}$, $p_{4}$, is defined in Equation (6).

(6) $$\rho =\theta +p_{2}\theta^{2}+p_{3}\theta^{3}+p_{4}\theta^{4}$$

where:

$$\theta =\frac{2}{\pi }\arctan\left(\frac{\sqrt{X^{2}+Y^{2}}}{Z}\right);\theta \in \left[0,1\right]$$

where: *X*, *Y* and *Z* are the coordinates of 3D point.

Thus, the pixel coordinates ($x_{d}$, $y_{d}$) for the projection of every 3D point can be calculated by the Equation (7):

(7) $$\left[\begin{array}{c} x_{d}\\ y_{d} \end{array}\right]=\left[\begin{array}{cc} C & D\\ E & F \end{array}\right]\left[\begin{array}{c} x_{hbt}\\ y_{hbt} \end{array}\right]+\left[\begin{array}{c} c_{x}\\ c_{y} \end{array}\right]$$

where:

$$\left[\begin{array}{c} x_{hbt}\\ y_{hbt} \end{array}\right]=\left[\begin{array}{c} \frac{\rho X}{\sqrt{X^{2}+Y^{2}}}\\ \frac{\rho Y}{\sqrt{X^{2}+Y^{2}}} \end{array}\right]$$

and $\left(c_{x},c_{y}\right)$ is the principal point in pixel coordinates.

**Figure 5:**
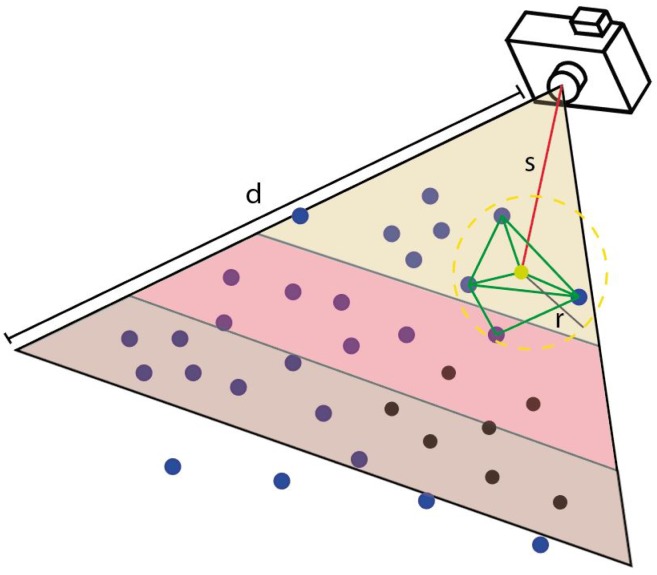
Occlusion detection on the point cloud. The target point (yellow) and their neighbors create a surface (green) to detect the occluded points (black).

In addition, a visibility test is carried out in order to detect the occlusion of shelf-hidden geometry in the model. In this regard, we propose a novel method to test the visibility of all objects from every camera position (Figure 5). Firstly, 3D points inside the view frustum of each camera are candidates to be projected in the image plane and to estimate their image coordinates (*x*,*y*). Secondly, these candidate points are ordered by the Euclidean distance between the camera and the position of viewpoint. Thirdly, from the nearest-to-farthest points, a minimal triangulated surface is formed by considering the target point and their neighbors. The neighbor search is based on the radius distance and the angle among normal vectors. Finally, the resulting surface is used to detect occluded points which are discarded in this capture. This algorithm is iterated until all points are checked.

Regarding the efficiency of the proposed method as well as the clustering process, which is described in the following section, the point cloud is spatially indexed using a kd-tree [31] in order to ensure an efficient performance. The value for *k* in the this spatial-data structure is determined on the three spatial coordinates (*X*, *Y*, *Z*) to reduce the consuming time for the neighbor search in the clustering process.

Finally, a weighting procedure is developed in order to assess the reliability of reflectance from each viewpoint. This algorithm considers the angle between the normal vector of the 3D point and the direction vector of the camera. The multispectral camera captures a more reliable reflectance of an object’s surface if its direction vector is similar to the normal vector of such object. If the angle between both vectors is greater, the reflectance detected is more irregular and less reliable. Our method focuses on the comparison between the vector direction of each multispectral view and the normal vector of every 3D point. In this way, reflectance maps are calculated considering three ranges: a perpendicular view ($0^{{}^{\circ}}$ to $25^{{}^{\circ}}$), an oblique view $25^{{}^{\circ}}$ to $60^{{}^{\circ}}$ and an indirect view (greater than $60^{{}^{\circ}}$).

This phase is very important in order to characterize the geometry with meaningful information about the spectral reflectance of the study area. Thus, the point cloud characterization makes the extraction of the existing materials in the captured scene easier. For each 3D point, we can determine the light which is reflected or absorbed for every observed multispectral band. The reflectance measurements, the RGB color and spatial location are processed like additional attributes.

### 2.5. Semantic Segmentation

In this section, the proposed methodology to identify different natural materials in the target area is described. Our approach is based on spatial and multispectral variables to develop the semantic segmentation of the point cloud. An innovative feature of this work, the point cloud has been enriched by the spectral reflectance observed from several multispectral bands. Therefore, the studied data contain multi-dimensions, which have been considered for the material extraction. Every 3D point is characterized by the spatial position in the scenario (coordinates: *X*, *Y*, *Z*), the reflectance in the green, red, REG and near-infrared bands, the vegetation index (NDVI) and the RGB color. Consequently, this detailed feature vector composed by eleven attributes is used for the semantic segmentation of the point cloud.

According to the above, the key differences between every natural entity have to be recognized in the target scenario. The relationships between these values and their variability are mainly determined by the properties of the materials. Therefore, the study of reflected light for each object lead to find out the distribution of different materials in the study scenario. In this paper, we propose a method for the semantic segmentation of homogeneous materials by considering the material appearance in the visible and non-visible range of the electromagnetic spectrum. For this purpose, a clustering algorithm is used to recognize every type of material without a labeled dataset. Unlike supervised learning, the clustering techniques are based on unsupervised learning since we do not have ground truth to compare the output of the clustering algorithm to the true labels and evaluate its performance.

In this study, our goal is to identify different types of materials real-life effects such as irregular lighting, hard or soft shadows, indirect illumination between objects, etc. To this end, a hierarchical cluster analysis [32,33] is developed to find out relatively homogeneous clusters. In contrast to the use of agglomerative clustering, which fails to detect local patterns in the data in local patterns without considering the global distribution of data, divisive clustering is also more accurate in making top-level partitioning decisions. This top-down approach builds the hierarchy on the assumption that all points are included in the same cluster. This cluster is split using a flat clustering algorithm. This procedure is recursively applied until a stopping criterion is introduced or each point is in a singleton cluster.

Our approach is based on the divisive clustering with an unknown number of clusters (*k*) to be detected in the point cloud. Initially, we do not have a previous knowledge of the classes of objects in the study scenario, therefore, the *k* value is undetermined. For each iteration of the algorithm, we aim to look for the two clusters which are more heterogeneous from each other. To this end, the most straightforward and generally accepted way to estimate the similarity between every object in a multidimensional space is to calculate Euclidean distances. The mathematical formulation is defined by the Equation (8). In this process, the kd-tree is used again to speed up the search for massive points during the step of neighbor search in the point cloud.

(8) $$d\left(p_{i},p_{j}\right)=\sqrt{\sum_{k=1}^{n}{\left(x_{ik}-x_{jk}\right)}^{2}}$$

where $p_{i}$ = ($x_{i1}$, $x_{i2}$, …, $x_{in}$) and $p_{j}$ = ($x_{j1}$, $x_{j2}$, …, $x_{jn}$) are two of *n* dimension points in point cloud *P*.

**Figure 6:**
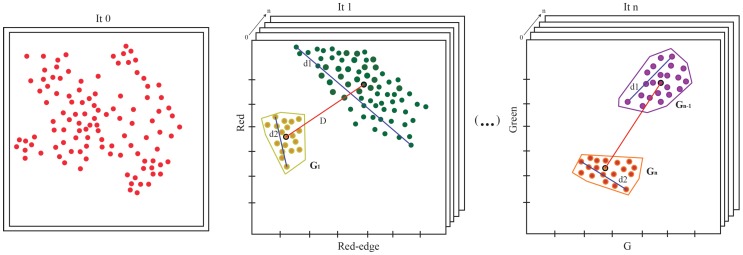
The main steps of the clustering algorithm.

Figure 6 presents a flow diagram of the proposed method. For each iteration, the K-means algorithm [34] is applied to extract the two most heterogeneous clusters. The main objective is to recognize the key feature pairs, which produce a greater heterogeneity of data.

The splitting criterion is determined by the maximization of the cluster diameter and the distance between the centroid of each cluster (Equation (9)). Initially, all points are considered into the same cluster. Then, a search of the pair of characteristics is carried out to detect the most significant difference between two groups. This process is recursively iterated until the stopping criterion is met. The condition of the loop end is when the cluster diameter is shorter than the distance between the centroids. For instance, in Figure 6, the diameter ($d_{2}$) of cluster $G_{1}$ is less than the distance (*D*) between the centroids, so this group cannot be subdivided again for the next iteration. Therefore, the heterogeneity of every cluster is highly reduced. In the final step, points are segmented into two groups $G_{n}$ and $G_{n-1}$, whose diameters are shorter than the distance between their centroids.

(9) $$Max_{x,y\in A}\left(d\left(x,y\right)\right)\;and\;Max_{x\in A,y\in B}\left(d\left(cx,cy\right)\right)$$

where *x* and *y* are points of the same cluster and *cx* and *cy* are the centroids of cluster *A* and *B* respectively.

## 3. Accuracy Assessment

The proposed method has been evaluated by measuring the accuracy of the process for the multispectral image mapping on the point cloud. On the one hand, the root-mean-square error (RMSE) is calculated to determine the quality of the alignment method between RGB and multispectral point clouds. The RGB point cloud is considered the reference model because it has a more precise geometry and a higher spatial resolution than the multispectral model. According to the result, the multispectral and RGB point clouds are automatically fixed in the same reference system. The error of this process is measured by applying the Equation (10).

(10) $$RMSE_{X,Y,Z}=\sqrt{\frac{1}{n}\Sigma_{i=1}^{n}{\left(X_{i,P_{1}}-X_{i,P_{2}}\right)}^{2}+{\left(Y_{i,P_{1}}-Y_{i,P_{2}}\right)}^{2}+{\left(Z_{i,P_{1}}-Z_{i,P_{2}}\right)}^{2}}$$

where (*X*, *Y* and *Z*) is the coordinates of a 3D point, $P_{1}$ is the RGB point cloud, $P_{2}$ is the multispectral point cloud and *n* the size of this last one.

**Figure 7:**
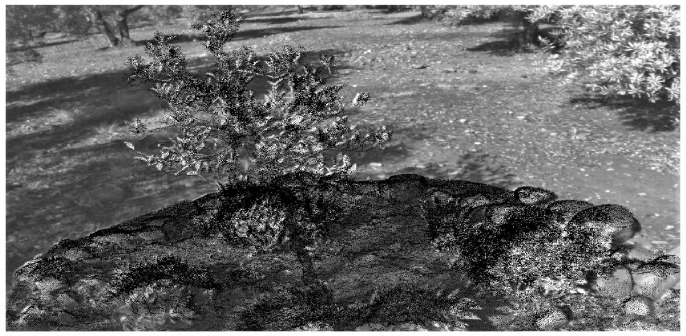
Image-based validation of inverse 3D projection.

On the other hand, the process of the multispectral image mapping on the RGB point cloud is checked by an image-based test. Figure 7 shows a multispectral image where pixels in the black color mean that these have been projected on the RGB model. The shape of every object captured in the study scene such as the tree, leaves or rocks can be correctly identified in the multispectral image. The background of the image is automatically discarded because it does not take part in the observed scenario.

## 4. Results

According to the application of the previous methodology, the observed study scenario can be classified by homogeneous materials with a similar appearance, which belong to different natural objects. The proposed framework demonstrates the contribution of multispectral images to characterize singular materials in nature. These results emphasize the potential of our application to generate a semantic segmentation of the point cloud by fusing spatial and spectral features.

### 4.1. Point Cloud Characterization

In this study, the first step is the geometric reconstruction of the study area. For this purpose, a photogrammetric process is performed by using the RGB images which were taken from multiple viewpoints. This point cloud presents a high spatial resolution (GSD = 0.07 cm). Moreover, multispectral images were captured at the same time in order to measure the spectral reflectance under natural lighting in several narrow bands. In this way, the target scene can be characterized by the spectral response of existing objects.

**Figure 8:**
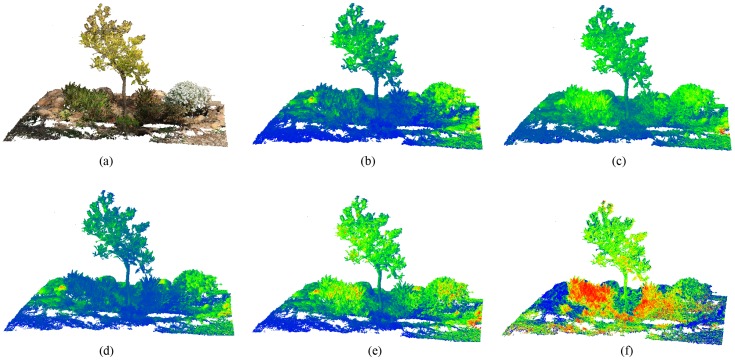
The studied observations of the point cloud: (**a**) RGB color; and the multispectral bands: (**b**) green, (**c**) NIR, (**d**) Red, (**e**) REG and (**f**) NDVI.

By applying the proposed method for multispectral image mapping on the point cloud, for each 3D point, we can inspect the spectral response on the 3D model. The error of the point cloud alignment is 0.85 cm, which can be tolerated in our approach because it is lower than the spatial resolution of the multispectral point cloud. Consequently, the point cloud is characterized by 11 attributes (X, Y, Z, R, G, B, red, green, REG, NIR, NDVI), which are used for the process of the point cloud segmentation. Figure 8 presents the enriched model for every information layer, which can be textured on the point cloud. The color scale is defined by the highest values in red color, the medium values in green and the lowest values in the blue color. On the RGB point cloud, blooming plants can be easily recognized but rocks and the tree trunk show a similar appearance as do the leaves and low vegetation. Regarding the highest values in the infrared domain (NIR and REG bands), these are shown by plants, where most of incident light is reflected. In the visible range, the reflectance detected in the vegetation area is lower than the rest due to the effect of chlorophyll pigments. Finally, in the NDVI point cloud vegetation areas can be clearly identified. However, this index is slightly saturated in the ground surface and the tree canopy. For every type of observation on the 3D model, some single entities can be clearly classified but not others. Consequently, the proposed divisible hierarchical clustering is carried out to find out the key feature patterns and extract the homogeneous materials in the study area.

### 4.2. Material Extraction

**Figure 9:**
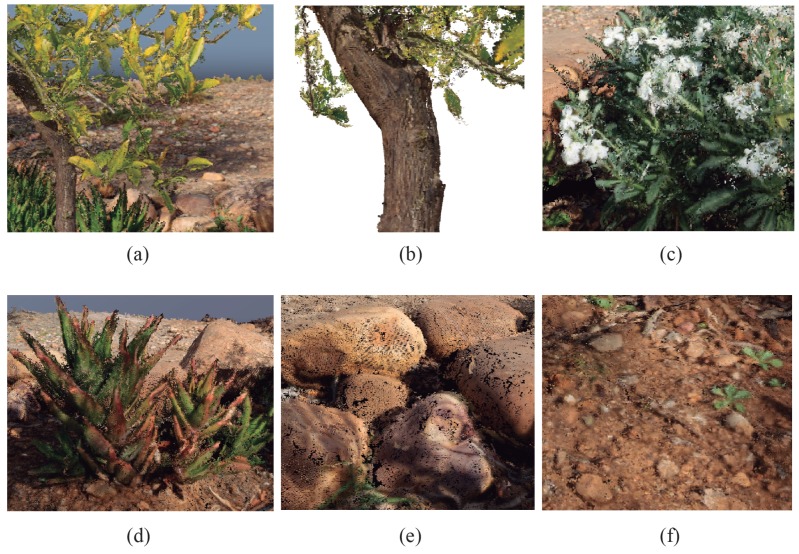
The identified materials in the scene. (**a**) leaves, (**b**) wood, (**c**) flowers, (**d**) plants, (**e**) rocks and (**f**) ground.

As a result of the application of the proposed segmentation of the point cloud, meaningful materials have been recognized in the study scenario. In the Figure 9, a detailed image of every identified material is shown. The leaves, the wood of the trunk, blooming plants, rocks, the ground and some shrubs have been detected in the point cloud.

**Figure 10:**
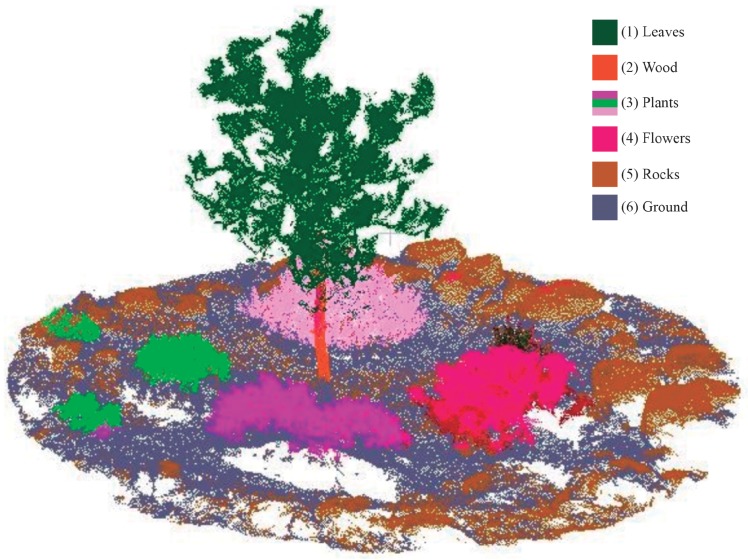
The semantic segmentation of natural materials in the point cloud.

According to the meaningful features used at different levels of the hierarchy, the NDVI and NIR were the first meaningful traits to detect leaves. Secondly, Red, REG and RGB values determined points of the tree trunk. Thirdly, NDVI, REG and Green characterized the shrubs whereas the blooming plants were influenced by NIR and Red bands. Fourthly, rocks were identified by combining the blue visible channel and the Red spectral band. Finally, the ground was recognized by the R and B visible channels and REG. Undoubtedly, all previous characteristics were combined with the spatial location of 3D points, which is considered a key constraint to limit the neighbor search in the point cloud. Figure 10 presents the resulting semantic point cloud, in which every cluster is identified as a single color. Moreover, the resulting dataset of the segmented point cloud is available in the Supplementary Material.

## 5. Discussion

This section presents a discussion regarding the results of the proposed approach. In this study, we propose a novel methodology to extract different materials in a natural space. The main goal for this work is to find out key differences between every object captured in the target scenario. Our approach is based on the unsupervised classification, so no previous labeled dataset is not required. Moreover, there is not a fixed number of clusters to be identified because this should change according to the heterogeneity of the study area. By applying our method, an enriched point cloud is generated as the result of the multispectral image mapping. This point cloud is segmented into different type of materials, which can be directly studied to understand the observed environment. Consequently, the results have a high impact and transferability to specific applications of remote sensing.

The material recognition on point clouds without training data is a challenging task. First, we focus on the point cloud reconstruction. The geometry presents a high spatial resolution less than one centimeter, so most object surfaces can be correctly modeled. Recently, the use of image-based approaches for 3D reconstruction has significantly increased because latest cameras are able to capture a high-resolution image and are more cost-effective than LiDAR system. However, the complex geometry as well as the homogeneous appearance of natural in the visible range objects arise some problems the semantic material-based segmentation. To characterize the observed scenario by other meaningful traits, a multispectral sensor was used for capturing four narrow bands. The result of the application of the proposed method for multispectral image mapping on the point cloud enable the fusion of the geometry and the reflected light for each 3D point on specific ranges of the electromagnetic spectrum. The variability of the spectral reflectance between every narrow band has been a key factor to recognize the properties of different natural materials. For instance, the visible light is highly absorbed by healthy vegetation whereas the infrared light is mostly reflected and rocks present a higher contrast in red and REG bands, etc. The NDVI is also considered an important feature because this index works properly for the recognition of vegetation materials. The collection of these heterogeneous attributes provided a comprehensive knowledge of the study scenario. As a result, six materials (leaves, tree trunk, plants, rocks and ground) were identified by applying the process of the semantic segmentation.

**Figure 11:**
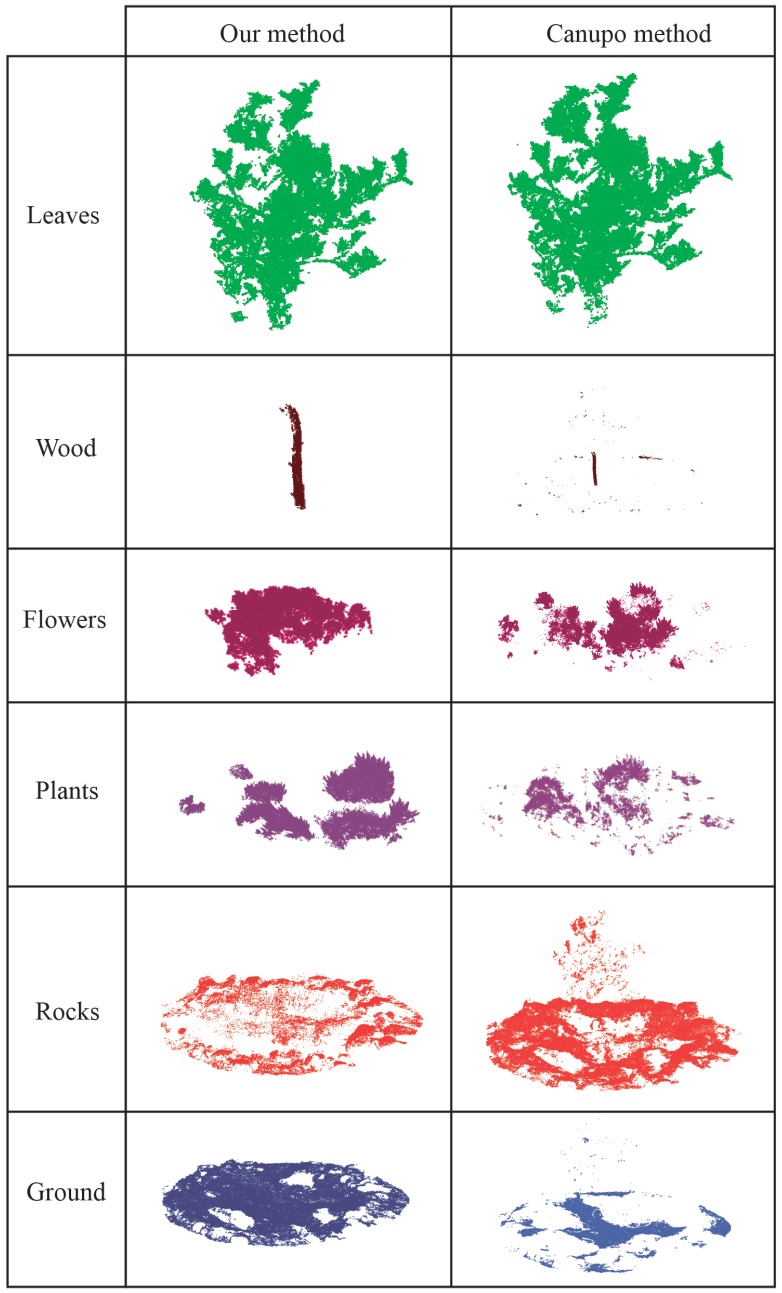
The results of our method and CANUPO algorithm.

In addition, our clustering method has been compared to the CANUPO suite [35]. It has been used by applications in geomorphology for the 3D point cloud segmentation of complex natural scenes using a multi-scale dimensional criterion. This algorithm uses a training dataset and extracts geometric features to characterize every class. An expert has to label the points on the 3D model, which compose the target material. According to this coarse labelling for every class, CANUPO takes some relevant characteristics to be assigned for this group of points by considering a confidence value. An important drawback of this method is that only binary classification can be performed. Moreover, it requires the human intervention for the point cloud labelling and a training dataset has to be created to enable the classification. A novelty of the proposed method lies in the fact that it does not need (1) a known number of classes to be recognized on the point cloud and (2) training datasets, which have to be tediously created. According to the results, Figure 11 reveals a more accurate recognition of natural material by the proposed method. Relevant differences can be observed for the segmentation of ground, rocks and plants. Flowers are also accurately identified by our method because these points present a higher reflectance in REG and NIR bands. Undoubtedly, the fusion of multispectral and spatial characteristics in the point cloud is essential to detect feature patterns associated to the behaviour of every natural material. Our results emphasize the possibility of recognizing different materials in a complex natural space by using 3D model enriched with multispectral images.

## 6. Conclusions

In this study, we have addressed the problem for the semantic segmentation of real-life materials, which present a similar appearance in nature. Scene understanding is a challenging task of the current research in computer vision. The automatic recognition of homogeneous natural materials on a point cloud without a previous labeled dataset is the main contribution of the proposed methodology. Our approach enables the detection of different type of materials by fusing spatial and spectral features of the study scenario. The main novelty is the application of a hierarchical divisive clustering by using an enriched photogrammetric 3D model, which has been characterized by the spectral reflectance observed from many multispectral images.

A custom rig camera was produced in order to integrate a high-resolution camera and a multispectral sensor, thus enabling the capture of multiple images by both devices from different viewpoints around the target scene. The study area was a controlled environment whose geometry was not too complex and contained some natural materials. Therefore, the proposed method could be validated and then, it could be applied for larger scenarios. Initially, the RGB images were used in the photogrammetric process to generate the point cloud. The resulting geometry had a high spatial resolution, 0.07 cm. Then, the multispectral sensor was used to measure the spectral reflectance in four narrow bands (Green, Red, NIR, REG). Moreover, NIR and Red bands were used together for the calculation of the NDVI. In this way, the material properties could be studied by observing the reflectance from every wavelength. By applying the proposed method, multispectral images can be correctly mapped on the point cloud, thus the 3D point is associated by a feature vector, which describes a more detailed behaviour of all objects captured. Our approach is based on heterogeneous data fusion on a 3D space. Consequently, we obtain a meaningful set of characteristics for the unsupervised classification of the point cloud.

According to this enriched point cloud, a hierarchical divisible clustering was proposed for the material recognition. In this study, we did not have any previous information about a fixed number of clusters in the dataset. Therefore, the clustering process did not require a previously labeled dataset as well as a known number of clusters to be classified. Moreover, the performance in clustering method was enhanced by the spatial indexing of a kd-tree structure. The results demonstrated the potential of our method to detect similar entities like rocks, the trunk, and ground as well as different type of vegetation materials. Our material-based segmentation was compared to the CANUPO algorithm based on a supervised classification. In spite of the fact that this method needs a manual labelling of target materials on the point cloud, our approach is able to get a more accurate identification of every material. Specifically, 3D points of rocks, the ground, the tree trunk can be correctly identified, whereas comparable methods can only detect the leaves and some parts of low vegetation.

In the future, our aim is the use of the proposed method to create an accurate ground-truth for using autoencoders and training 3D neural networks. We hope to extend our data structure to propose a more realistic physically-based rendering of natural environments using the Graphics Processing Unit (GPU). In addition, we would like to test our method for the recognition of urban materials.

## Supplementary Materials

The following are available online at https://www.mdpi.com/1424-8220/20/8/2244/s1.
